# Association between hemoglobin glycation index with insulin resistance and carotid atherosclerosis in non-diabetic individuals

**DOI:** 10.1371/journal.pone.0175547

**Published:** 2017-04-20

**Authors:** Maria Adelaide Marini, Teresa Vanessa Fiorentino, Elena Succurro, Elisabetta Pedace, Francesco Andreozzi, Angela Sciacqua, Francesco Perticone, Giorgio Sesti

**Affiliations:** 1 Department of Systems Medicine, University of Rome Tor Vergata, Rome, Italy; 2 Department of Medical and Surgical Sciences, Viale Europa, University Magna-Græcia of Catanzaro, Catanzaro, Italy; Virgen Macarena University Hospital, School of Medicine, University of Seville, SPAIN

## Abstract

Hemoglobin glycation index (HGI), defined as the difference between the observed HbA1c value and the value of HbA1c predicted from plasma glucose levels, represents a measure of the degree of non-enzymatic glycation of hemoglobin and it has been found to be positively associated with micro- and macro-vascular complications in subjects with type 2 diabetes. To investigate the pathophysiological abnormalities responsible for the increased cardiovascular risk of patients with higher HGI, we evaluated the association of HGI with cardio-metabolic characteristics in nondiabetic offspring of type 2 diabetic individuals. Insulin sensitivity, measured by a hyperinsulinemic-euglycemic clamp, cardio-metabolic risk factors including lipid profile, uric acid and inflammatory factors, and ultrasound measurement of carotid intima–media thickness (IMT) were assessed in 387 nondiabetic individuals. Participants were stratified in tertiles according to HGI (high, moderate and low). As compared with subjects with low HGI, those with high HGI displayed an unfavorable cardio-metabolic risk profile having significantly higher values of BMI, waist circumference, triglycerides, uric acid, fasting insulin, inflammatory markers, such as high sensitivity C reactive protein, erythrocytes sedimentation rate, complement C3, fibrinogen, and white blood cell count, and carotid IMT, and lower HDL and insulin-stimulated glucose disposal. In a linear regression analysis model including several atherosclerotic risk factors such as gender, age, BMI, inflammatory factors, lipid profile, insulin-stimulated glucose disposal, fasting insulin, uric acid, and blood pressure, HGI was the major predictor of IMT (*β* = 0.35; *P* = 0.001). In a logistic regression analysis adjusted for confounders, individuals with high HGI showed a 2.7-fold increased risk of vascular atherosclerosis (OR 2.72, 95%CI 1.01–7.37) as compared with subjects with low HGI. The present findings support the notion that HGI may be a useful tool to identify a subset of nondiabetic individuals conceivably harboring a higher risk of cardiovascular disease.

## Introduction

Glycated hemoglobin (HbA1c) test is an indirect measure of average glycemia over approximately the previous 3 months that has been recommended not only for monitoring glucose control among persons with diabetes but also as a diagnostic test for both type 2 diabetes and conditions of increased risk of diabetes (the so-called prediabetes) [1]. Compared with fasting glucose or 2-h post load glucose values, HbA1c has some advantages as a diagnostic test since it has higher repeatability and pre-analytical stability, it can be measured in the nonfasting state, and has less day-to-day perturbations during illness conditions. In addition, HbA1c has been shown to be a better predictor of cardiovascular disease than fasting plasma glucose even in nondiabetic populations [2,3]. Nonetheless, discordances between HbA1c values and other measures of glycemic control have been observed [4–7]. Indeed, inter-individual variations in HbA1c caused by factors other than blood glucose concentration have been reported in subjects with type 2 diabetes [8–10], and in nondiabetic individuals [11–18]; these variants were likely due to genetic factors [15,16] or to differences in hemoglobin glycation rates or in red cell survival among different ethnic groups [17,18].

A statistical method to measure the disparity between actual HbA1c and the predicted value of HbA1c based on plasma glucose levels has been developed and termed hemoglobin glycation index (HGI) [19–22]. HGI is calculated as the difference between the observed HbA1c value and the predicted HbA1c derived by inserting the individual fasting plasma glucose concentration into a population regression equation expressing the linear association between HbA1c and plasma glucose levels [19,22]. HGI may help to identify diabetic subjects with a greater risk of micro- and macro-vascular complications [21,22]. Applying HGI analysis to the Diabetes Control and Complications Trial (DCCT), it has been observed that type 1 diabetic patients with a high HGI exhibited a greater risk for retinopathy and nephropathy [21]. Accordingly, in patients with type 2 diabetes participating to the Action to Control Cardiovascular Risk in Diabetes (ACCORD) trial, a higher HGI value was found to be associated with retinopathy and nephropathy at baseline, and with greater mortality in the intensive treatment group [22].

The question arises whether disparities between HbA1c and fasting plasma glucose as assessed by HGI may unveil a different cardio-metabolic risk profile within nondiabetic individuals.

To gain a deeper insight into the pathophysiological abnormalities responsible for the increased risk of cardiovascular complications observed in type 2 diabetic patients with higher HGI [22], we evaluated the associations of HGI with cardio-metabolic characteristics including insulin sensitivity, assessed by the hyperinsulinemic-euglycemic clamp technique, lipid profile, inflammatory factors, and carotid intima-media thickness (IMT) in a cohort of non-diabetic offspring of type 2 diabetic individuals.

## Materials and methods

### Study subjects

Two different samples of White nondiabetic adults (≥18 years of age) were studied.

Sample 1 comprises 2,055 nondiabetic individuals consecutively recruited at the Department of Systems Medicine of the University of Rome-Tor Vergata and at the Department of Medical and Surgical Sciences of the University “Magna Graecia” of Catanzaro [23]. This sample was used to estimate the linear relationship between fasting plasma glucose and HbA1c in the study population to calculate the predicted HbA1c value [22]. The inclusion criteria were: age ≥20 years, absence of diabetes, defined as HbA1c ≥6.5% or fasting plasma glucose ≥126mg/dl, and presence of one or more cardio-metabolic risk factors including elevated blood pressure, dyslipidemia, overweight/obesity, and family history for diabetes. Exclusion criteria encompassed: history of any malignant disease, end stage renal disease, heart failure, gastrointestinal diseases associated with bleeding or malabsorption, autoimmune diseases, acute or chronic infections, acute or chronic pancreatitis, haemoglobinopathies including beta thalassemia trait, erythrocyte disorders, accumulation diseases such as amyloidosis and hemochromatosis, history of drug abuse, self-reporting alcohol consumption of >20 g/day, positivity for antibodies to hepatitis C virus (HCV) or hepatitis B surface antigen (HBsAg), treatments able to modulate glucose metabolism, including lipid-lowering and hypoglycemic agents, corticosteroids, and use of antiplatelet or anticoagulant medications.

Sample 2 comprises 387 non-diabetic offspring subjects participating in the European Network on Functional Genomics of Type 2 Diabetes (EUGENE2) project [24] who had only one parent with type 2 diabetes. Subjects were consecutively recruited at the Department of Medical and Surgical Sciences of the University ‘Magna Graecia’ of Catanzaro and at the Department of Systems Medicine, University of Tor Vergata, Rome as previously described [25]. Participants underwent anthropometrical evaluation including measurements of body mass index (BMI), waist circumference, body composition evaluated by bioelectrical impedance, and three consecutive measurements of clinic blood pressure were obtained in the sitting position, after five minutes of quiet rest. Resting heart rate was measured in the morning with subjects in the supine position, after at least 30 min of quiet rest, by electrocardiography. After 12-h fasting, a 75 g OGTT was performed with sampling for plasma glucose and insulin determinations. Insulin sensitivity was assessed by euglycemic hyperinsulinemic clamp study, as previously described [26]. Briefly, a priming dose of insulin (Humulin, Eli Lilly & Co., Indianapolis, IN) was administrated during the initial 10 min to acutely raise plasma insulin followed by continuous insulin infusion fixed at 40 mU/m^2^ x min. The blood glucose level was maintained constant during the 2-h clamp study by infusing 20% glucose at varying rates according to blood glucose measurements obtained with a glucose analyzer at 5 minute intervals (mean coefficient of variation of blood glucose was < 5%). Glucose disposal (M) was calculated as the mean rate of glucose infusion measured during the last 60 min of the clamp examination (steady-state) and it is expressed as milligrams per minute per kilogram fat-free mass (M_FFM_) measured with the use of electrical bioimpedance. High resolution B-mode ultrasound was used to measure IMT of the common carotid artery using an ATL HDI 3000 ultrasound system (Advanced Technology Laboratories, Bothell, WA) equipped with a 7.5 MHz transducer, as previously described [27]. A value of IMT >0.9 mm was used as index of vascular atherosclerosis according to the 2013 Guidelines for the management of arterial hypertension released by the Task Force for the Management of Arterial Hypertension of the European Society of Hypertension (ESH) and of the European Society of Cardiology (ESC) [28].

The protocol was approved by Institutional Ethics Committees and written informed consent was obtained from all participants in accordance with principles of the Declaration of Helsinki.

### Analytical determinations

HbA1c was measured with high performance liquid chromatography using a National Glycohemoglobin Standardization Program (NGSP) certified automated analyzer (Adams HA-8160 HbA1C analyzer, Menarini, Italy). Total and high lipoprotein density (HDL) cholesterol, triglycerides, and glucose levels were assayed by enzymatic methods (Roche, Basel, Switzerland). Serum insulin concentrations were determined with a chemiluminescence-based assay (Immulite®, Siemens, Italy). Serum uric acid was measured by the URICASE/POD method implemented in an autoanalyzer (Boehringer Mannheim, Mannheim, Germany). High sensitivity C reactive protein (hsCRP) levels were assessed by an automated instrument (CardioPhase® hsCRP, Milan, Italy). White blood cell count was determined using an automated particle counter (Siemens Healthcare Diagnostics ADVIA® 120/2120 Hematology System, Italy). Erythrocytes sedimentation rate (ESR) was measured automatically by the stopped-flow technique in a capillary microphotometer (Alifax Test 1 System Polverara, Italy). Fibrinogen and complement C3 were measured by an automated nephelometric technology using the BN™II System analyzer (Siemens Healthcare, Italy).

### Statistical analysis

Variables with skewed distribution including triglycerides, fasting insulin, hsCRP, and ESR were natural log transformed for statistical analyses. Continuous data are expressed as means ± SD. Categorical variables were compared by χ^2^ test. Anthropometric and metabolic differences between groups were assessed after adjusting for age, gender and BMI using a generalized linear model with post hoc Fisher's least significant difference correction for pairwise comparisons. A multivariable linear regression analysis was performed in order to evaluate the independent contribution of HGI and other cardio-metabolic risk factors to IMT. A logistic regression analysis adjusted for confounders was used to determine the association between the study groups and vascular atherosclerosis (IMT >0.9 mm) [28]. A *P* value <0.05 was considered statistically significant. All analyses were performed using SPSS software programme Version 17.0 for Windows.

## Results

### Calculation of HGI

**Fig 1:**
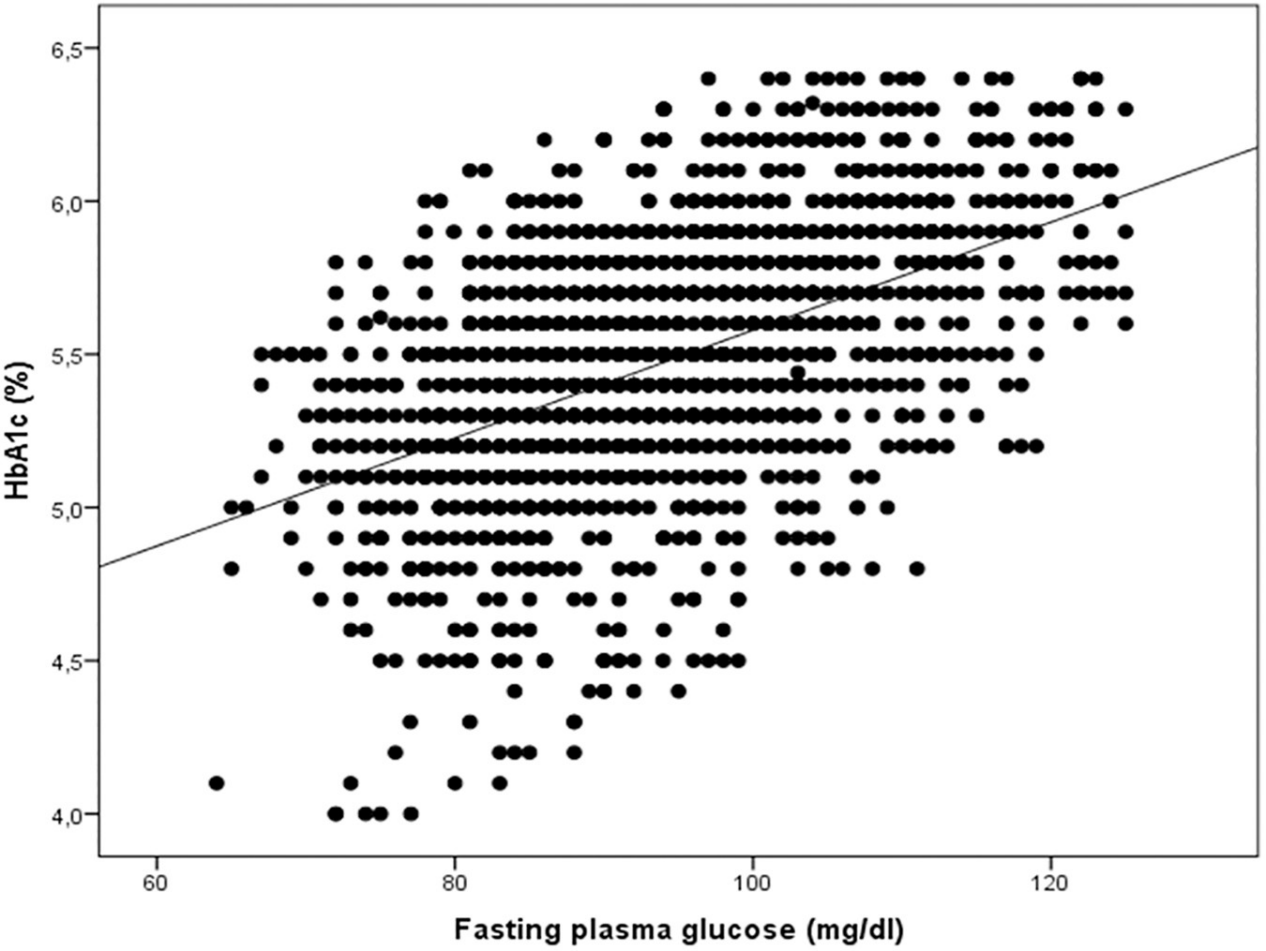
Linear relationship between HbA1c and fasting plasma glucose in the study sample 1.

Sample 1 consisting of 2,055 nondiabetic adult individuals (mean age 47.8±14.6 years) was used to estimate the linear relationship between fasting plasma glucose and HbA1c. As shown in Fig 1, fasting plasma glucose and HbA1c were highly correlated (*r* = 0.55) in a cohort with widely varying glycemic control: mean HbA1c was 5.4 ±0.4%, and fasting plasma glucose 93±11 mg/dl. The degree of correlation is very similar to the one observed in previous studies in type 2 diabetic individuals [22]. Despite the good correlation, it is evident that there is a substantial scatter as well. Next, predicted HbA1c was calculated for 387 non-diabetic offspring subjects included in sample 2 by inserting fasting plasma glucose concentration into the sample 1 linear regression equation (HbA1c = 0.0158 * fasting glucose levels (mg/dl) +4.0311). HGI was calculated by subtracting the predicted value of HbA1c from the observed HbA1c levels, as previously described [22]. Study participants were stratified into tertiles (low, moderate, or high HGI groups) according to their HGI value. The use of a tertile classification system is consistent with previous HGI studies [22].

### Cardio-metabolic characteristics

The mean age of the whole study sample was 37±10 years, 530 (47%) individuals were male, and mean BMI was 29±6 kg/m^2^. Anthropometric and metabolic features of the study sample 2 stratified according to tertiles of HGI value are shown in Table 1. No significant differences between the three study groups were observed with respect to gender. Subjects with high HGI were older and heavier than individuals with low or moderate HGI. By design, individuals with high HGI exhibited higher values of HbA1c, and lower concentrations of fasting plasma glucose; however, no differences in 2h-post load glucose levels were observed amongst the three study groups.

**Table 1:** Anthropometric and metabolic characteristics of the study subjects stratified according to HGI tertiles.

Variables	Low HGI (1)	Moderate HGI (2)	High HGI (3)	*P*	*P* 1 vs. 2	*P* 1 vs. 3	*P* 2 vs. 3
*n* (Male/Female)	129 (51/78)	129 (41/88)	129 (55/74)	0.18	0.24	0.70	0.09
Age (*yr*)	36±10	37±10	39±10	0.01*	0.32*	0.004*	0.07*
BMI (*kg/m*^*2*^)	29.0±7.6	29.5±7.4	32.9±8.6	0.001**	0.77**	<0.0001**	0.001**
Waist circumference (*cm*)	94±17	947±16	103±18	<0.0001**	0.94**	<0.0001**	<0.0001**
SBP *(mmHg)*	121±13	121±18	124±17	0.31	0.37	0.12	0.50
DBP *(mmHg)*	77±9	78±11	79±11	0.12	0.81	0.10	0.06
HbA1c (%) [*mmol/mol*]	4.9±0.3 [30 mmol/mol]	5.3±0.2 [34 mmol/mol]	5.8±0.3 [40 mmol/mol]	<0.0001	<0.0001	<0.0001	<0.0001
Fasting glucose (*mg/dl*)	93±11	90±9	88±11	<0.0001	<0.0001	<0.0001	0.74
2-h glucose (*mg/dl*)	113±29	114±31	119±34	0.18	0.96	0.10	0.11
Fasting insulin *(μU/ml)*	10±5	11±7	15±9	0.005	0.89	0.005	0.003
Total cholesterol (*mg/dl*)	190±39	193±38	198±39	0.86	0.98	0.62	0.64
HDL (*mg/dl*)	53±13	52±13	48±11	0.07	0.70	0.03	0.08
Triglycerides (*mg/dl*)	102±52	103±58	134±82	0.03	0.96	0.02	0.02
Uric acid (*mg/dl*)	4.70±1.30	4.85±1.27	5.18±1.49	0.02	0.10	0.006	0.23
hsCRP (*mg/l*)	1.5±1.7	2.7±3.1	5.0±5.0	<0.0001	<0.0001	<0.0001	0.07
Fibrinogen (*mg/dl*)	274±68	286±62	309±70	0.002	0.27	0.001	0.01
Complement C3 (*g/l*)	1.07±0.18	1.15±0.22	1.25±0.23	0.07	0.22	0.02	0.22
ESR (*mm/h*)	12±11	14±11	16±13	0.02	0.16	0.008	0.15
White blood cell count (*x10*^*9*^*/l*)	6358±1763	6800±2130	7698±2069	<0.0001	0.06	<0.0001	0.002
Insulin-stimulated glucose disposal (*mg/min x Kg FFM*)	10.6±3.7	10.5±4.4	8.4±4.7	0.03	0.89	0.02	0.02
Intima-media thickness (*mm*)	0.69±0.13	0.70±0.13	0.77±0.14	0.03	0.81	0.03	0.01
Vascular atherosclerosis (OR 95%CI)	1 (Reference)	1.15 (0.37–3.57)	2.72 (1.01–7.37)				

Data are means ± SD. Triglycerides, hsCRP, ESR, fasting, 1-h and 2-h insulin were log transformed for statistical analysis, but values in the table represent a back transformation to the original scale. Categorical variables were compared by χ^2^ test. Comparisons between the three groups were performed using a generalized linear model with post hoc Fisher's least significant difference correction for pairwise comparisons. *P* values refer to results after analyses with adjustment for age, gender, and BMI.

*P values refer to results after analyses with adjustment for gender.

**P values refer to results after analyses with adjustment for gender, and age. BMI = body mass index; SBP = systolic blood pressure; DBP = diastolic blood pressure; HDL = high density lipoprotein; hsCRP = high sensitivity C-reactive protein; ESR = erythrocyte sedimentation rate.

After adjusting for age, gender and BMI, subjects with high HGI displayed higher levels of triglycerides, uric acid, and fasting plasma insulin, and lower HDL cholesterol and insulin-stimulated glucose disposal in comparison with individuals with low HGI. Additionally, after adjusting for age, gender and BMI, subjects with high HGI displayed a significant increase of all the inflammatory markers measured i.e. hsCRP, ESR, complement C3, fibrinogen and white blood cell count in comparison with individuals with low HGI.

Atherosclerosis can be detected noninvasively in preclinical stages by measuring carotid IMT, a well-established measure of early atherosclerosis that is largely utilized as a surrogate marker for cardiovascular disease [29].

To estimate the independent contributor of HGI to IMT levels, we performed a linear regression analysis in a model including gender, age, BMI, systolic and diastolic blood pressure, total and HDL cholesterol, triglycerides, inflammatory markers, uric acid, fasting insulin and insulin sensitivity, estimated by the insulin-stimulated glucose disposal. Comparison of standardized coefficients allowing the determination of the relative strength of the association of each variable with IMT (listed from strongest to weakest) is reported in Table 2. HGI was the major contributor to IMT (*β* = 0.35; *P* = 0.001) followed by hsCRP (*β* = 0.34; *P* = 0.007), ESR (*β =* 0.28; *P* = 0.02), and age (*β* = 0.17; *P =* 0.05). The full model explained 28% of IMT variation.

**Table 2:** Multiple regression analysis evaluating IMT as dependent variable.

Dependent variable	Independent contributors	Coefficient β	*P*
IMT	HGI hsCRP ESR Age BMI Gender DBP White blood cell count Triglycerides Fibrinogen Total cholesterol Fasting insulin HDL SBP Complement C3 Insulin-stimulated glucose disposal Uric acid	0.35 0.34 0.28 0.17 0.20 0.19 0.16 0.07 0.05 0.03 -0.06 -0.06 -0.04 -0.03 -0.03 0.006 0.002	0.001 0.007 0.02 0.05 0.11 0.11 0.26 0.43 0.74 0.76 0.59 0.61 0.71 0.84 0.74 0.96 0.98

Linear regression analysis in a model including gender, age, BMI, systolic and diastolic blood pressure, total and HDL cholesterol, triglycerides, inflammatory markers, uric acid, fasting insulin, and insulin sensitivity, estimated by the insulin-stimulated glucose disposal.

IMT = intima-media thickness; BMI = body mass index; HGI = hemoglobin glycation index; hsCRP = high sensitivity C-reactive protein; ESR = erythrocyte sedimentation rate; SBP = systolic blood pressure; DBP = diastolic blood pressure; HDL = high density lipoprotein.

**Fig 2:**
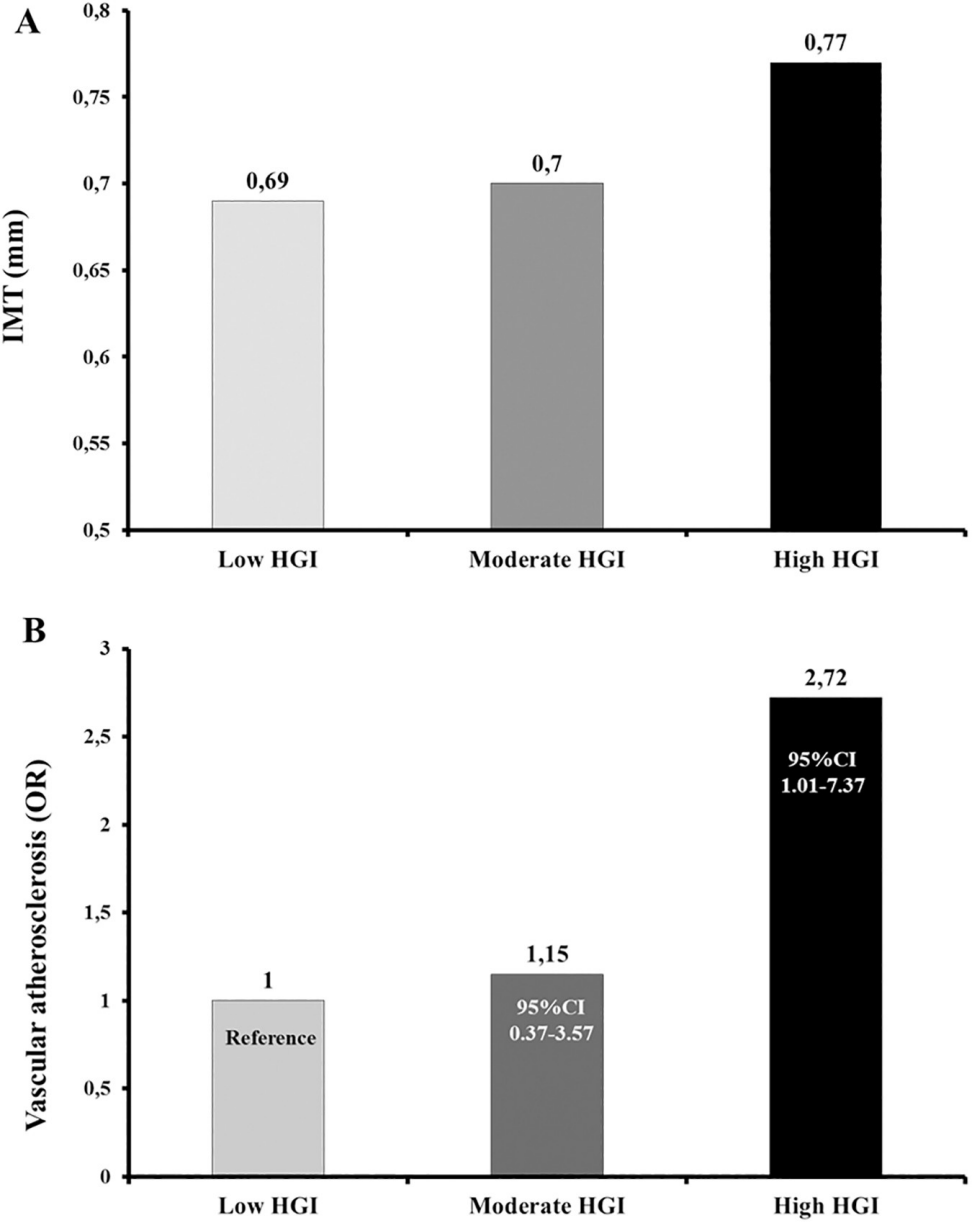
IMT values (A), and OR 95%CI for vascular atherosclerosis, defined as IMT >0.9 (B), in the study sample 2 stratified according to tertiles of HGI.

After adjusting for age, gender, and BMI subjects with high HGI displayed a significant increase in IMT as compared with individuals with low HGI (Table 1 and Fig 2A). A logistic regression analysis adjusted age, gender, and BMI was used to determine the association between the HGI values and subclinical vascular atherosclerosis, defined as IMT >0.9 mm according to the ESH/ESC guidelines [28]. Subjects with high HGI showed a 2.7-fold increased risk of having vascular atherosclerosis (OR 2.72, 95%CI 1.01–7.37) as compared with subjects with low HGI (Table 1 and Fig 2B). When in the logistic regression model HGI values were analyzed as continuous variable rather than a categorical variable (tertiles), we observed that each unit increase in HGI value was associated with a 5.2-fold increased risk of having vascular atherosclerosis (OR 5.20, 95%CI 1.84–14.72).

## Discussion

The risk of developing micro- and macro-vascular complications of both type 1 and type 2 diabetes is closely related to the chronic level of plasma glucose [30,31]. HbA1c is an integrated measure of mean glycemia over the preceding 2–3 months, and is considered as the gold standard for measurement of glycemic control in subjects with diabetes. In addition, HbA1c is a very good predictor of cardiovascular disease even in nondiabetic populations [2,3], and recently, the ADA recommended the adoption of the HbA1c test for the diagnosis of diabetes and prediabetes [1]. It is well established that HbA1c levels result from a posttranslational modification of hemoglobin by glucose, and that the main factor influencing the rate of glycation in vivo is the prevailing concentration of plasma glucose. Nonetheless, discordances between HbA1c levels and other measures of glycemic control have been reported [4–7]. Indeed, a number of individuals exhibit consistently higher or lower HA1c levels than those that would be anticipated on the basis of fasting plasma glucose [22, 32], mean blood glucose (self-monitored) [19,33] or continuous glucose monitoring [34, 35]. Differences in the glycation of hemoglobin between individuals with the same fasting plasma glucose values can be assessed by the calculation of HGI [22,32]. HGI is a measure of the disagreement between the observed value of HbA1c and the one predicted on the basis of blood glucose levels. Individuals with low and high HGI have HbA1c levels that are lower or higher than predicted, respectively, compared with other individuals with similar blood glucose levels. It has been shown that higher HGI is associated with increased risk of developing nephropathy and retinopathy in patients with type 1 diabetes [21, 36], and with a greater risk of diabetic complications, hypoglycemia and total mortality in a subgroup of the ACCORD population [22].

Overall, these data support the idea that a higher degree of non-enzymatic glycation of intracellular proteins may play a pathogenic role in micro- and macro-vascular complications related to hyperglycemia. If this is the case, it is conceivable that a higher HGI affecting intracellular protein glycation process may increase the individual cardio-metabolic risk burden among nondiabetic individuals. In the present study, we investigated the association between HGI with insulin sensitivity, assessed by the gold standard euglycemic hyperinsulinemic clamp, and carotid IMT, a validated measure of early stage of atherosclerosis [28,29], in a Caucasian cohort of nondiabetic offspring of type 2 diabetic patients. Impaired insulin sensitivity is considered an important risk factor for atherosclerotic disease [37–39], and a key determinant of cardiovascular risk factors, including visceral obesity, atherogenic dyslipidemia, and hypertension, clustering within the metabolic syndrome [40]. We found that individuals with high HGI exhibited a worse metabolic risk profile including higher whole body and visceral adiposity, triglycerides, uric acid, fasting insulin, and lower HDL cholesterol and insulin-stimulated glucose disposal in comparison with individuals with low HGI, independently of confounders such as age and gender. Notably, no significant differences in post-load glucose concentrations were observed between the study groups, suggesting that the association between HGI and cardo-metabolic risk factors were independent of other measures of glucose homeostasis. Furthermore, we found a significant association between higher HGI and inflammatory status as subjects with high HGI displayed increased levels of inflammatory biomarkers including hsCRP, complement C3, ESR, fibrinogen, and white blood cell count in comparison to individuals with low HGI independently of age, gender, and BMI. As a result of the higher cardio-metabolic risk burden, subjects with high HGI presented a significant increase in carotid intima media thickness, a well validated indicator of subclinical atherosclerosis.

The mechanism by which elevated HGI are associated with increased risk of vascular atherosclerosis is unsettled. One way for non-enzymatic glycosylation to cause both insulin resistance and vascular atherosclerosis is by fostering advanced glycation end products (AGEs) [41–43]. Notably, in subjects with diabetes increased HGI has been associated with higher levels of AGEs [44]. AGEs interacting with their receptors (RAGE) on the cell membrane can damage target cells by several mechanisms including altered function of intracellular proteins modified by AGEs, activation of nuclear factor κ-B, causing a raise in gene expression of inflammatory mediators, and increased production of reactive oxygen species [41,42]. There is evidence in animal models that oral advanced AGEs induce insulin resistance by altering insulin receptor signaling leading to impaired glucose-uptake [45]. Moreover, in subjects with type 2 diabetes, it has been shown that baseline serum AGEs correlate with fasting insulin, and indexes of insulin resistance, and an AGE-restricted diet for 4-months improves insulin sensitivity [46]. Accordingly, we observed that individuals with high HGI had lower insulin sensitivity and consequently higher levels of fasting insulin as compared with individuals with low HGI independently of age, gender, and BMI.

In addition, chronic sub-clinical inflammation could be a unifying mechanistic factor linking elevated HGI with vascular atherosclerosis. We found that individuals with higher HGI displayed increased levels of a cluster of inflammatory markers including hsCRP, ESR, fibrinogen, white blood cell count, and complement C3 suggesting that a greater degree of non-enzymatic glycation may play a pathogenic role in inducing chronic sub-clinical inflammation. Indeed, compelling evidence suggests that RAGE has a considerable role in innate immunity [47], and inflammatory response is known to play a role in the development of atherosclerotic cardiovascular disease [48,49]. It has been observed that subjects with diabetes treated with a low AGEs diet for 6 weeks exhibit a significant reduction of serum AGEs levels and markers of inflammation as hsCRP, and TNFα [50], thus reinforcing the role of AGEs in promoting sub-clinical inflammation. Moreover, it has been reported that RAGEs are upregulated in the atherosclerotic plaques of subjects with type 2 diabetes, and their overexpression is associated with enhanced inflammatory reaction [50–53]. Because HGI has been shown to reflect the burden of AGEs in the tissues, it is conceivable that activation of RAGE system may play a role in the increased risk of vascular atherosclerosis observed in individuals with high HGI.

The present study has several strengths including the use of the gold standard hyperinsulinemic euglycemic clamp for insulin sensitivity assessment, the demographically homogeneous group of Italian subjects from European ancestry comprising both men and women, wealth of detailed clinical, anthropometric and biochemical variables collected by trained professionals according to a standardized protocol, the exclusion of confounding conditions potentially affecting red cell turnover, such as anemia and major blood loss, the exclusion of subjects treated with corticosteroids, lipid-lowering and anti-hypertensive drugs, the centralization of laboratory analyses, the use of OGTT and HbA1c data to carefully exclude type 2 diabetes, the use of a rigorously standardized HbA1c assay, and the ultrasound measure of carotid IMT performed by an experienced examiner who was blinded to the clinical and biochemical data of the participants.

Nevertheless, some limitations should be considered in the interpretation of the present results. A first limitation of the study is that each diagnostic test including HbA1c and OGTT was only performed once. Although such an approach reflects clinical practice, and is common in epidemiological studies, the intra-individual variability of glucose parameters cannot be taken into account, and some individuals might have been misclassified. In addition, the observed differences in cardio-metabolic risk factors may be, in part, due to differences in age and BMI between the HGI groups; however, all comparisons between groups were adjusted for these potential confounders. Another limitation of the present study is its cross-sectional design, making causal interpretations of associations between higher non-enzymatic glycation of intracellular proteins assessed by HGI and risk of cardiovascular disease difficult. Indeed, the current results reflect only an association with early atherosclerosis and not incident cardiovascular disease. Moreover, it can also be argued that our results might have been affected by the presence of a family history of type 2 diabetes. However, type 2 diabetes and cardiovascular disease share common genetic determinants, and many individuals who develop cardiovascular disease have a family history of diabetes. Furthermore the study sample used to estimate the linear relationship between fasting plasma glucose and HbA1c displayed a mean age higher than the one observed in the analysed cohort. It has been demonstrated that HbA1c levels were positively associated with age in subjects without diabetes [54]. However in consideration that a 0.014- and 0.010-unit increase in HbA1c per year has been descried in non-diabetic individuals participating to the Framingham Offspring Study and National Health and Nutrition Examination Survey, respectively, we believe that the impact of the difference in the age distribution between the two study samples (about 10 years) in HGI calculation was marginal. Finally, caution in generalizing these results is warranted since the current results are only based on White individuals, and could not be extendible to other ethnic groups. Indeed, previous studies have shown that HbA1c levels are higher among Blacks, Hispanics, American Indians, and Asian Americans compared to Whites likely due to differences in hemoglobin glycation or red cell survival [17,55].

In conclusion, the present findings support the notion that HGI may be a useful tool to identify a subset of nondiabetic individuals conceivably harboring a higher risk of cardiovascular disease.
